# Activity of Antimicrobial Peptides and Ciprofloxacin against *Pseudomonas aeruginosa* Biofilms

**DOI:** 10.3390/molecules25173843

**Published:** 2020-08-24

**Authors:** Muhammad Yasir, Debarun Dutta, Mark D.P. Willcox

**Affiliations:** 1 School of Optometry and Vision Science, University of New South Wales, Sydney, NSW 2052, Australia; d.dutta@aston.ac.uk (D.D.); m.willcox@unsw.edu.au (M.D.P.W.); 2 Optometry and Vision Science Research Group, Aston University, Birmingham B4 7ET, UK

**Keywords:** antibiotic resistance, antimicrobial peptides, ciprofloxacin, *P. aeruginosa*, biofilm, combination therapy

## Abstract

*Pseudomonas aeruginosa* is increasingly resistant to conventional antibiotics, which can be compounded by the formation of biofilms on surfaces conferring additional resistance. *P. aeruginosa* was grown in sub-inhibitory concentrations of the antimicrobial peptides (AMPs) melimine and Mel4 or ciprofloxacin for 30 consecutive days to induce the development of resistance. Antibiofilm effect of AMPs and ciprofloxacin was evaluated using crystal violet and live/dead staining with confocal microscopy. Effect on the cell membrane of biofilm cells was evaluated using DiSC(3)-5 dye and release of intracellular ATP and DNA/RNA. The minimum inhibitory concentration (MIC) of ciprofloxacin increased 64-fold after 30 passages, but did not increase for melimine or Mel4. Ciprofloxacin could not inhibit biofilm formation of resistant cells at 4× MIC, but both AMPs reduced biofilms by >75% at 1× MIC. At 1× MIC, only the combination of either AMP with ciprofloxacin was able to significantly disrupt pre-formed biofilms (≥61%; *p* < 0.001). Only AMPs depolarized the cell membranes of biofilm cells at 1× MIC. At 1× MIC either AMP with ciprofloxacin released a significant amount of ATP (*p* < 0.04), but did not release DNA/RNA. AMPs do not easily induce resistance in *P. aeruginosa* and can be used in combination with ciprofloxacin to treat biofilm.

## 1. Introduction

Biofilms are multi-layered, localized, organized and heterogenous networks of microorganisms surrounded by self-produced extracellular polymeric substances (EPS) containing biomolecules such as polysaccharides, proteins, nucleic acids, and lipids [1,2]. Biofilms cause 80% of human bacterial infections, including biomaterial-associated infections, lung infections in cystic fibrosis patients and wound infections [3,4]. These biofilm-associated infections are difficult to treat with antibiotics due, amongst other things, to the interference of EPS with the penetration of antibiotics into the biofilms. Moreover, the growth rate of bacteria in biofilms causes them to become tolerant to antibiotics [5,6]. These factors can increase the antibiotic resistance of biofilm-associated microbes by up to 500–5000 times [7,8]. Biofilm properties further complicate the eradication of the biofilm infection, leading to the development of chronic infections. Often the only solution to this problem is the removal of the infected device or tissue, or the use of large amounts of antibiotics [9]. However, this increases treatment costs, due to the need for revision surgery, and the potential cytotoxicity of the large amounts of antibiotics [10]. The importance of biofilm-related infections in the clinical setting and their inherent resistance to conventional antibiotic treatment urgently demands the development of compounds that can disrupt biofilms.

Some antimicrobial peptides (AMPs) are known to have strong activity against multidrug-resistant bacterial biofilms [11]. The antibiofilm activity of certain AMPs can be increased by using them together with conventional antibiotics [12]. These combined treatments are becoming an important part of treating biofilm-related infections, such as chronic wounds or biomaterial-associated infections caused by *P. aeruginosa* [13]. Combination treatments can target bacteria in different metabolic states, at low pH and under conditions of limited oxygen or nutrients [14]. AMP-based combination treatments can work at decreased concentrations of the antimicrobials and extend the spectrum of activity of the individual components. This may reduce the chances of cytotoxicity, and combinations may reduce resistance development. The mechanism of action of many AMPs alone involves the disruption of cell membranes. However, when used in combination, they may act as carrier peptides for transporting antibiotics across membranes into the cytoplasm and so increase the effectiveness of antibiotics by increasing intercellular uptake [15].

AMPs have been recognized as promising alternatives for conventional antibiotics due to their rapid bacterial killing and multiple target sites in bacteria [16]. Due to these properties, it is believed that microbes may not easily become resistant to AMPs. Melimine (TLISWIKNKRKQRPRVSRRRRRRGGRRRR) is a cationic chimeric peptide of two naturally occurring peptides melittin and protamine [17]. Melimine has a wide spectrum of activity targeting clinical isolates of Gram-negative and Gram-positive bacteria (including methicillin-resistant *S. aureus* MRSA, and multi-drug-resistant *P. aeruginosa*), fungi and protozoa, such as *Acanthamoeba* [17,18]. Moreover, it is not cytotoxic at well above active concentrations [17,18] and causes hemolysis of horse red blood cells lysis at concentrations 15 times higher than its minimum inhibitory concentration MIC [19]. A derivative of melimine called Mel4 (KNKRKRRRRRRGGRRRR) is active against planktonic *P. aeruginosa* [20]. It is non-cytotoxic to mammalian cells in vitro and causes hemolysis at concentration 64 times higher than its MIC [17,18,19]. Moreover, it is non-cytotoxic in animal model studies and in human clinical trials [21,22]. The ability of *P. aeruginosa* to develop resistance to a shorter version of melimine, Mel4, is not known. Melimine and Mel4 can synergize with ciprofloxacin against the planktonic forms of *P. aeruginosa* [23], but it is not known if they are active against biofilms. In the current study, the potential of *P. aeruginosa* to develop resistance against melimine, Mel4 and ciprofloxacin, and the ability of these AMPs to reduce biofilms was evaluated.

## 2. Results

### 2.1. Minimal Inhibitory Concentration and Minimal Bactericidal Concentration

The MICs and minimal bactericidal concentrations MBCs of AMPs and ciprofloxacin are given in (Table 1). The MIC of Mel4 against *P. aeruginosa* strains 6294, 6206 and ATCC 19660 was 62.5 µg/mL. Melimine had its lowest MIC of 125 µg/mL against *P. aeruginosa* ATCC 27853. For all strains, the MBC was usually 2× the MIC except for strain 6294 where the MBC for both melimine and Mel4 was ≥4× the MIC, while for ATCC 19660 the MBC for Mel4 was equivalent to the MIC. Ciprofloxacin had its lowest MIC of 0.25 µg/mL against *P. aeruginosa* 6206 and highest MIC of 1 µg/mL against *P. aeruginosa* ATCC 19660. For all strains, the MBC of ciprofloxacin was usually 2× the MIC except for strain 6294 where the MBC for was equivalent to the MIC.

### 2.2. Development of Resistance to AMPs and Ciprofloxacin

**Figure 1:**
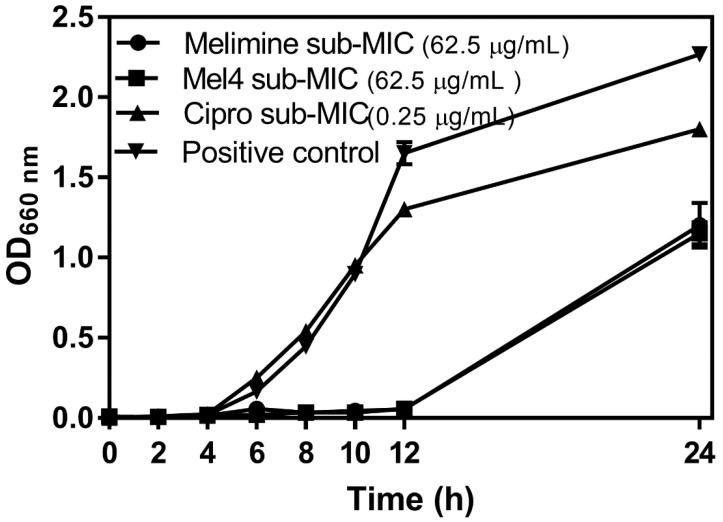
Growth curves for *P. aeruginosa* ATCC 27853 at sub-MIC of the antimicrobial peptides (AMPs) melimine and Mel4 or ciprofloxacin (Cipro). The positive control was growth in the absence of any antimicrobial.

The growth curves of *P. aeruginosa* ATCC 27853 at sub-MICs of melimine, Mel4 or ciprofloxacin over 24 h are presented in Figure 1. The initial growth of *P. aeruginosa* ATCC 27853 at its sub-MIC for ciprofloxacin (Figure 1) was similar to growth without the antimicrobial, but the final amount of bacteria was reduced. There was a much longer lag phase when *P. aeruginosa* was grown at sub-MICs of melimine or Mel4 and the amount of bacteria that grew after 24 h was also reduced. There was no difference in growth characteristics at sub-MICs of melimine or Mel4.

**Figure 2:**
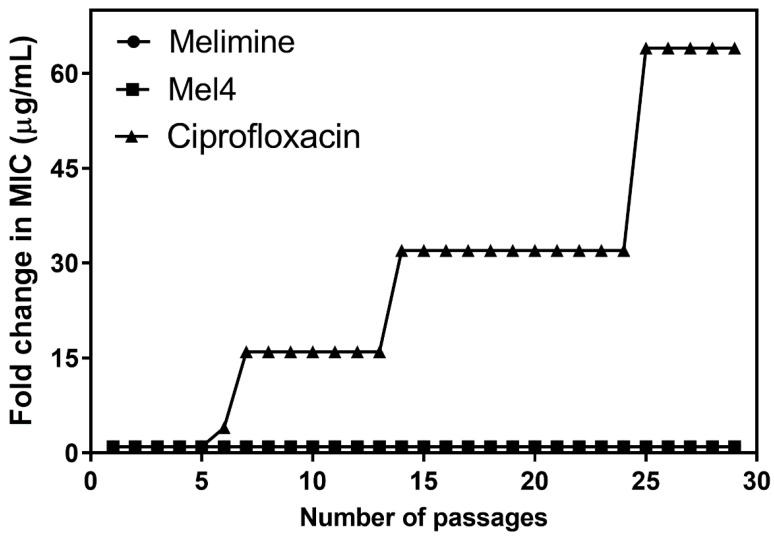
Change in resistance of *P. aeruginosa* ATCC 27853 to ciprofloxacin, melimine or Mel4 following serial passage at sub-MIC for 30 days. The MIC of melimine and Mel4 did not change, and values overlap at the bottom of the figure.

Changes in MICs of *P. aeruginosa* ATCC 27853 after exposure to sub-MICs of melimine, Mel4 or ciprofloxacin over 30 days are presented in Figure 2. The MICs of melimine and Mel4 did not change over time, suggesting a limited potential of resistance development to these peptides (Figure 2). Compared to the peptides, there was a rapid development of resistance to ciprofloxacin. Resistance developed to ciprofloxacin after seven days of serial passage with an initial 4-fold increase in MIC. The MIC increased 32-fold after 15 passages and to 64-fold after 30 passages (Figure 2).

### 2.3. Inhibition of Biofilm Formation by AMPs and Ciprofloxacin Alone or in Combination

**Figure 3:**
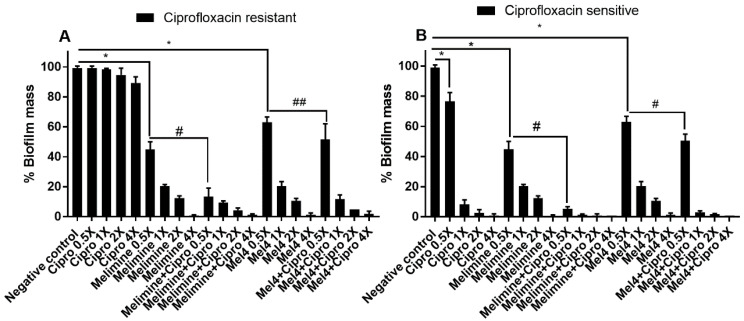
Inhibition of biofilm formation of *P. aeruginosa* ATCC 27853. Biofilm formation of the ciprofloxacin-resistant (**A**) or sensitive (**B**) cells of *P. aeruginosa* ATCC 27853 was inhibited by various concentrations of melimine, Mel4 and ciprofloxacin alone or in combination. The strain was made resistant to ciprofloxacin by sub-passage for 30 days at a sub-MIC concentration (Section 4). * represent significant (*p* < 0.001) decreases compared to the negative control (bacteria grown in the absence of antibiotics). # indicates significant (*p* < 0.001) decrease for the combinations compared to melimine or Mel4 alone while ## indicates *p* = 0.051 compared to Mel4 alone. Means (±SD) of three independent repeats in triplicate. Negative control = bacteria grown in the absence of antimicrobials, Cipro = ciprofloxacin.

Ciprofloxacin alone did not significantly inhibit the biofilm formation of the ciprofloxacin-resistant cells of *P. aeruginosa* ATCC 27853 even at 4× MIC (Figure 3A; *p* = 0.209). Melimine and Mel4 inhibited the biofilm formation of the ciprofloxacin-resistant cells at 0.5× MIC by 55% and 37% respectively (*p* < 0.001). Melimine and ciprofloxacin in combination at 0.5× MIC produced 87% inhibition of biofilm which was significantly greater (*p* < 0.001) than the 48% produced by the Mel4 and ciprofloxacin combination at 0.5× MIC (Figure 3A). The combined effect of Mel4 and ciprofloxacin was not significantly different from Mel4 alone at 0.5× (*p* = 0.051), but reached significance at 1× MIC (Figure 3A). The ciprofloxacin sensitive cells of *P. aeruginosa* ATCC 27853 behaved in a similar manner to the resistant cells, with the exception that biofilm formation was significantly inhibited at 0.5× MIC and above of ciprofloxacin alone, and there was a significant effect of Mel4 and ciprofloxacin compared to Mel4 alone at 0.5× MIC (Figure 3B).

### 2.4. Disruption of Pre-Formed Biofilms by AMPs and Ciprofloxacin Alone or in Combination

**Figure 4:**
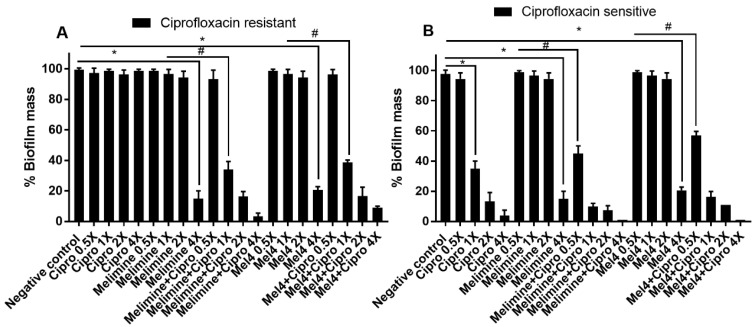
Disruption of pre-established biofilm of *P. aeruginosa* ATCC 27853. Biofilms of the ciprofloxacin-resistant (**A**) and sensitive (**B**) cells of *P. aeruginosa* ATCC 27853 were disrupted at various concentrations by melimine, Mel4 and ciprofloxacin alone or in combination. * represents significant (*p* < 0.001) decrease compared to the negative control (biofilm treated with buffer). # indicates significants (*p* < 0.001) decrease for the combinations compared to melimine or Mel4 alone. Means (±SD) of three independent repeats in triplicate. Negative control = bacteria grown in the absence of antimicrobials. Cipro = ciprofloxacin.

The antibiofilm potential of AMPs and ciprofloxacin alone or in combination against performed (24 h) biofilms of ciprofloxacin-resistant and sensitive isolates of *P. aeruginosa* ATCC 27853 is shown in Figure 4. Ciprofloxacin did not reduce pre-formed biofilms of the ciprofloxacin-resistant cells of *P. aeruginosa* ATCC 27853 at any of the concentrations tested (*p* > 0.999; Figure 4A). Both AMPs could not disrupt the biofilm of the ciprofloxacin-resistant cells at 1× and 2× MICs. However, they were effective at 4× their MICs, disrupting more than 79% of pre-formed biofilms compared to the control in the absence of antimicrobials (*p* < 0.001). At 4× MIC, the reduction in biofilm produced by both the AMPs was similar (*p* > 0.999). The combination of melimine and ciprofloxacin at 1× MIC caused 66% (*p* < 0.001) biofilm disruption compared to melimine alone at 1× MIC. Similarly, the combination of Mel4 and ciprofloxacin eradicated 61% biofilm at 1× MIC compared to Mel4 alone at 1× MIC (*p* < 0.001). The combination of either melimine or Mel4 with ciprofloxacin eradiated 83% biofilm at 2× MIC (*p* < 0.001).

Ciprofloxacin began to significantly reduce pre-formed biofilms at 1×, 2× and 4× MICs compared to control (*p* < 0.001; Figure 4B), producing 65%, 87% and 96% reduction in biofilms, respectively. Melimine and Mel4 were active at 4× MICs, reducing the pre-formed biofilm by 79% (*p* < 0.001). When AMPs were used with ciprofloxacin, the antibiofilm effect was enhanced, and the combined treatment was able to disrupt pre-formed biofilms at lower concentrations. The combination of melimine and ciprofloxacin caused 55% biofilm disruption at 0.5× MIC and 90% disruption at 1× MIC compared to control (*p* < 0.001; Figure 4B). Mel4 and ciprofloxacin displayed similar antibiofilm activity, disrupting 43% and 84% biofilm at 0.5× and 1× MIC, respectively (Figure 4B). The combined treatment of either AMP with ciprofloxacin at 1× MIC or 2× MICs resulted in similar biofilm disruption (*p* > 0.999).

### 2.5. Visualization of Biofilms

**Figure 5:**
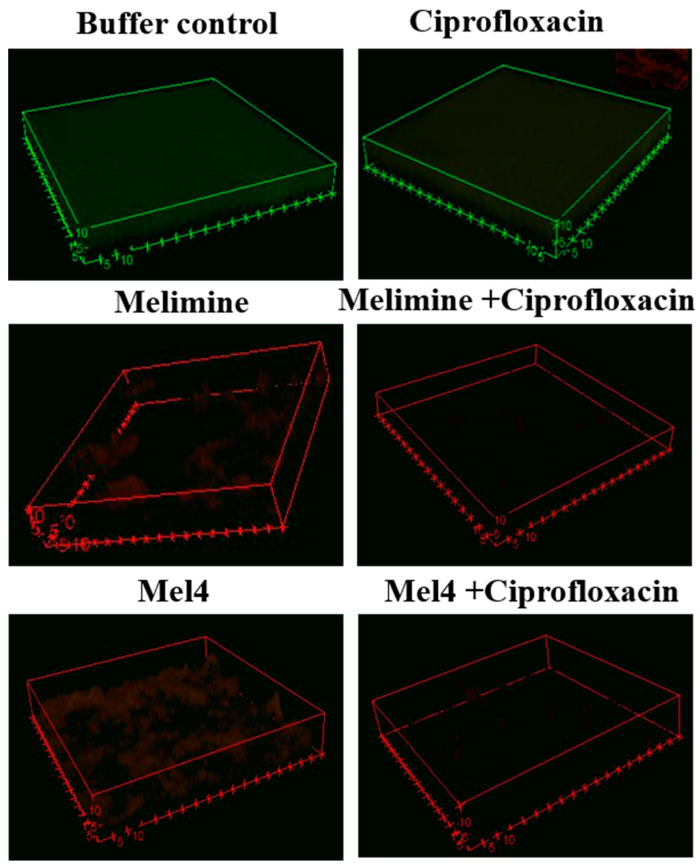
Representative confocal laser scanning microscopy images of biofilms of the ciprofloxacin-resistant isolate of *P. aeruginosa* ATCC 27853 after treatment with AMPs and ciprofloxacin alone or in combination. The antibiofilm effects were evaluated at 4× the MIC of all antimicrobials after incubation for 24 h. The biofilms of *P. aeruginosa* were stained with SYTO-9 (excited at 488 nm, green live cells) and propidium iodide (excited at 514 nm, red dead cells). The cells exposed to ciprofloxacin alone when excited at 514 nm had a red-ish color indicating some of the cells had taken up the propidium iodide, which was confirmed in the insert showing the biofilm excited at 514 nm.

Biofilms treated with buffer (HEPES) or ciprofloxacin alone had an overall dimension of 60 × 60 × 7 µM (Figure 5). The biofilms exposed to buffer were mainly green indicating that they were alive, whereas the biofilms exposed to ciprofloxacin had a red-ish color indicating that some of the cells in the biofilm had died (Figure 5). Biofilms treated with melimine or Mel4 at 4× their MICs had less biofilm mass with dimensions of 53 × 53 × 5 µM and the cells were mainly stained red indicating many dead cells. Very sparse biofilms were seen for the combinations of melimine and ciprofloxacin, or Mel4 and ciprofloxacin at 4× MICs (Figure 5).

### 2.6. Cell Membrane Depolarization

**Figure 6:**
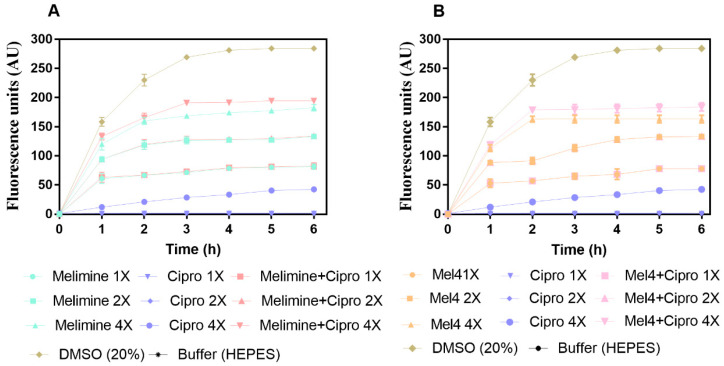
Cell membrane depolarization of pre-formed (24 h) biofilm cells. Cell membrane depolarization of *P. aeruginosa* ATCC 27853 (**A**) by melimine and ciprofloxacin alone or in combination, and (**B**) by Mel4 and ciprofloxacin alone or in combination against pre-formed (24 h) biofilms. Means (±SD) of three independent repeats in triplicate. Cipro = ciprofloxacin, DMSO = dimethyl sulfoxide.

The results for cell membrane depolarization of *P. aeruginosa* ATCC 27853 is presented in Figure 6. Melimine and Mel4 depolarized the cell membrane of *P. aeruginosa* in a concentration and time-dependent manner (Figure 6A,B). Both peptides depolarized the cell membrane of biofilm cells within 1 h of incubation at 1×, 2× and 4× MICs. The fluorescence intensity produced as the result of the release of the DiSC3(5) dye was greater at 4× than 2× or 1× concentrations for either melimine or Mel4 (*p* < 0.001). The rate of release of the dye increased up to 2 h and became constant thereafter for all concentrations. Ciprofloxacin did not cause cell membrane depolarization at 1× and 2× MIC over the entire duration of the experiment. However, at 4× MIC of ciprofloxacin there was significantly higher (*p* < 0.001) fluorescence compared to control but significantly less (*p* > 0.999) compared to melimine or Mel4 at 1× or 2× their MIC after 2 h incubation.

The combined membrane depolarizing effect of melimine or Mel4 with ciprofloxacin was similar to the individual effects of melimine or Mel4 at their corresponding 1×, 2× and 4× MICs (*p* > 0.999; Figure 6A,B). At 4× MIC, the melimine and ciprofloxacin combination caused greater membrane depolarization than the Mel4 and ciprofloxacin combination after 1h of incubation (*p* < 0.031). The positive control (DMSO 20%) reached a maximum fluorescence at 3 h, and this became constant thereafter.

### 2.7. ATP Leakage and DNA/RNA Release

**Figure 7:**
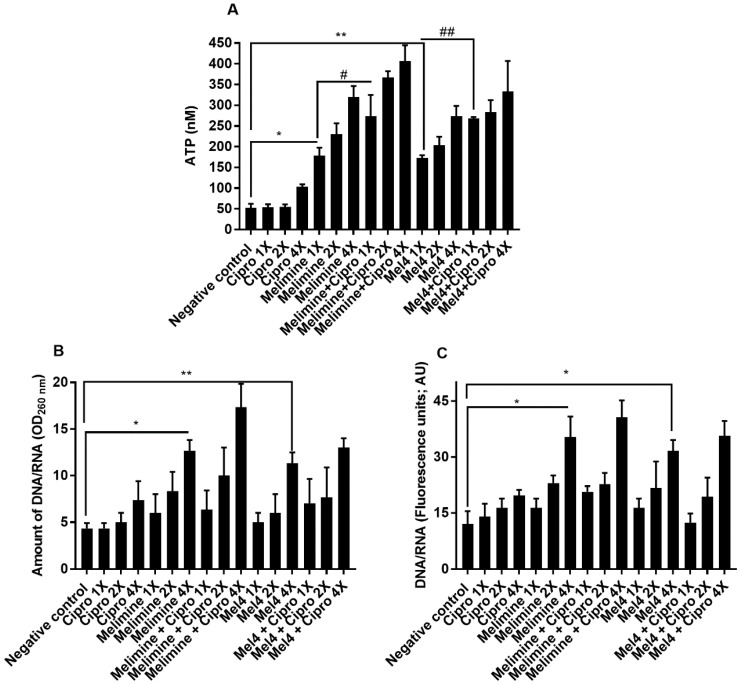
Leakage of (**A**) ATP and (**B**,**C**) nucleic acid from pre-formed biofilm cells of *P. aeruginosa* ATCC 27853. Leakage of ATP or nucleic acid from pre-formed (24 h) biofilms of *P. aeruginosa* ATCC 27853 following treatment for 3 h with either of the two peptides and ciprofloxacin alone or in combination. The strain was made resistant to ciprofloxacin by passage for 30 days at a sub-MIC concentration (0.25 µg/mL). * represent significant (*p* = 0.001) and ** represent significant (*p* = 0.002) increases in the amount of ATP or nucleic acid release compared to the negative control. # represent significant (*p* = 0.044) increase in the release of ATP of the combination of melimine and ciprofloxacin compared to melimine alone, and ## represent a significant (*p* = 0.039) increase in the release of ATP of the combination of Mel4 and ciprofloxacin compared to Mel4 alone.

Incubation with both AMPs released a substantial amount of ATP from pre-formed biofilms of *P. aeruginosa* ATCC 27853 in a concentration-dependent manner (Figure 7A). Melimine at 1×, 2× and 4× MIC induced leakage of 178 ± 19 nM, 230 ± 26 nM and 320 ± 26 nM ATP, respectively, compared to buffer treated negative control (*p* ≤ 0.001). Mel4 at 1×, 2× and 4× MICs released 172 ± 7 nM, 203 ± 21 nM, 273 ± 25 nM extracellular ATP, respectively, compared to buffer (*p* ≤ 0.002). The amount of ATP released by melimine and Mel4 at their corresponding MICs was similar (*p* ≥ 0.999). The addition of ciprofloxacin alone to pre-formed biofilms did not result in the release of extracellular ATP at any of the concentrations tested (*p* > 0.999; Figure 7A), even though there appeared to be a slight increase when 4× MIC was added. The combination of melimine and ciprofloxacin released significantly greater amounts of ATP (*p* = 0.044; 273 ± 51 nM) than was released by melimine (178 ± 19 nM) alone. There was a similar effect on ATP leakage from the combination at 2× and 4× MICs. The combination of Mel4 and ciprofloxacin at 1×, 2× and 4× concentrations induced leakage of 268 ± 3 nM, 283 ± 29 nM, 333 ± 74 nM ATP, respectively (Figure 7A). At 1× MIC, the combination of Mel4 and ciprofloxacin released significantly greater amounts of ATP (*p* = 0.039) than was released by Mel4 alone. Both the melimine and ciprofloxacin or Mel4 and ciprofloxacin combination had similar effects at their corresponding MICs (*p* > 0.999).

The release of nucleic acids (260 nm absorbing material) after incubation for 4 h with the antimicrobials from pre-formed biofilms of *P. aeruginosa* ATCC 27853 is shown in Figure 7B. Incubation with melimine resulted in 13 ± 1 times (*p* < 0.001) and Mel4 increased 11 ± 1 times (*p* = 0.012) nucleic acid from pre-formed biofilms at 4× MIC only compared to control. There was no difference in the amount of nucleic acid released after incubation with melimine or Mel4 at 4× their MICs (*p* > 0.999). Incubation with ciprofloxacin did not cause significant nucleic acid leakage from the pre-formed biofilms at any concentration tested (*p* > 0.999; Figure 7B). The combination of melimine and ciprofloxacin resulted in greater amounts (17 ± 3 times; *p* ≤ 0.001) of nucleic acid release than buffer treated cells. Incubation with melimine alone or combination with ciprofloxacin released a similar amount of nucleic acid at 4× MIC (*p* = 0.705). Similarly, at 4× MIC, Mel4 alone or in combination with ciprofloxacin released a similar amount of nucleic acid (*p* > 0.999). There was no significant difference between melimine or Mel4 alone or in combination with ciprofloxacin at 4× MICs (*p* > 0.999). Melimine and Mel4 either alone or with ciprofloxacin combination resulted in the release of greater amounts of nucleic acid labelled by Sytox green at 4× MIC than any other concentration of AMPs or ciprofloxacin used alone or in combination at their corresponding MICs (Figure 7C). This showed comparable results to the assay that measured release at 260 nm.

## 3. Discussion

Sub-MICs of antimicrobials are known to generate resistant mutants by triggering reactions, such as bacterial adaption to stress and biofilm formation. These can result in increased resistance to antibiotics and disinfectants, resulting in potential therapeutic failure [24,25,26]. In the current experiment, *P. aeruginosa* ATCC 27853 was unable to become resistant to melimine or Mel4 at their sub-MICs, whereas growth of this strain in a sub-MIC of ciprofloxacin caused high levels of resistance to develop over 30 days. Biofilms that are resistant to ciprofloxacin were reduced by using a combination of melimine or Mel4 with ciprofloxacin either when biofilms were forming or following maturation. Melimine and Mel4 acted on the membranes of biofilm cells, and this resulted in the death of cells and release of intracellular material.

*P. aeruginosa* 27853 developed resistance to ciprofloxacin rapidly but could not develop resistance to the AMPs melimine or Mel4. Resistance to ciprofloxacin in *P. aeruginosa* occurs, due to mutation in topoisomerase IV or overexpression of a chromosomally encoded bacterial efflux pump which leads to increased transport of ciprofloxacin out of the bacterial cell [27,28]. The inability of *P. aeruginosa* to develop resistance against melimine and Mel4 may be due to the rapid killing kinetics of these peptides and their action on cell membranes [20]. Bacteria appear to rarely gain resistance to AMPs that target bacterial membranes. [25]. However, resistance to polymyxin (B or E) has been observed in *P. aeruginosa*, and this can be due to the acquisition of genes rather than mutations [29]. *P. aeruginosa* can reduce the net negative charge on its anionic cell envelope molecule lipid A to resist the action of AMPs [29]. Perhaps these structures remained unaltered in response to melimine and Mel4.

Biofilm formation is a mechanism which can also protect microbial cells from antibiotics [30]. Melimine and Mel4 prevented biofilm formation of *P. aeruginosa* at a concentration lower than their MICs. This is similar to LL-37 which could also inhibit *P. aeruginosa* biofilm formation at sub-MIC [31]. The inhibition of biofilm by AMPs has been shown to be the result of stimulating twitching motility, influencing quorum sensing or degrading signaling molecules, such as ppGpp [32,33], or decreasing the expression of polysaccharide-intercellular adhesin genes [34]. The mechanism of action of melimine and Mel4 inhibiting biofilms of *P. aeruginosa* appeared to be very similar to their mode of action on *P. aeruginosa* cells in suspension [20], that is disrupting cell membranes resulting in release in intracellular contents. Further studies should be conducted to evaluate whether melimine and Mel4 also act on twitching motility, quorum sensing or signaling molecules, such as ppGpp.

Both AMPs could reduce the formation of biofilm by ciprofloxacin-resistant and sensitive strains at concentrations lower than their MICs. Inhibition of biofilm formation by ciprofloxacin was only observed for the ciprofloxacin sensitive strain. Similar to a previous report [35], pre-formed biofilms of *P. aeruginosa* were more resistant to ciprofloxacin, and pre-formed biofilms of *P. aeruginosa* are more resistant to antimicrobials than immature biofilms [36]. The pre-formed biofilms in the current study, regardless of the sensitivity of planktonic cells to ciprofloxacin, were more resistant to ciprofloxacin and both of the peptides. However, the combined use of melimine or Mel4 and ciprofloxacin lowered the active concentrations of each antimicrobials resulting in significant biofilm disruption of the ciprofloxacin sensitive strain at its sub-MIC. In contrast, biofilms of the ciprofloxacin-resistant strain required 1× MIC of these combinations to be significantly affected. The combination of AMPs and ciprofloxacin resulted in greater inhibition of biofilm at 0.5× MIC than either alone, suggesting that both the peptides have synergistic activity with ciprofloxacin against *P. aeruginosa* which is in agreement with a previous report [23]. Another report showed that the AMP 1018 synergizes with ceftazidime, ciprofloxacin, imipenem, or tobramycin and inhibits biofilms of *P. aeruginosa*, *E. coli*, *Acinetobacter baumannii* at 0.5× MIC [37].

Melimine and Mel4 could also disperse pre-formed biofilms, although higher concentrations were needed for this than to inhibit the formation of biofilms. Disruption of pre-formed biofilms by the AMPs D,L-K6L9, Seg5L, Seg5D, Seg6L, and Seg6D peptides have been shown to be due to direct killing the biofilm cells and degrading the biofilm mass [38]. Melimine and Mel4 killed cells in biofilms, as shown by the higher proportion of PI positive (stained red = dead cells) cells after incubation. Application of either AMP also reduced the biofilm mass compared to buffer treated controls. Previously, immobilized melimine and Mel4 killed adherent *P. aeruginosa* cells and reduced total adhered cells [39].

Both melimine and Mel4 killed *P. aeruginosa* biofilm cells probably by disrupting the cell membranes, which resulted in the loss of cellular integrity as seen by release of ATP and DNA/RNA [40]. These mechanisms of action against *Pseudomonas* biofilm cells were similar to the mechanisms against planktonic cells [20]. The ability of the AMP/ciprofloxacin combinations to disrupt pre-formed biofilms might be related to AMPs facilitating higher intracellular uptake of ciprofloxacin [41]. Perhaps the damage to cell membranes caused by melimine and Mel4 allowed greater access for ciprofloxacin into the bacteria. AMPs permeabilize the cell membrane and enter either alone or penetrate together with antibiotic and interrupt the intracellular metabolic functions [42,43]. Polymyxin B and Gramicidin S disrupt membrane structural and functional proteins of *P. aeruginosa* and allowed entry of ciprofloxacin into cells [44].

## 4. Materials and Methods

### 4.1. Peptides Synthesis

Melimine and Mel4 were synthesized by conventional solid-phase peptide protocols [45,46] and were procured from Auspep Peptide Company (Tullamarine, Victoria, Australia). The purity of the peptides was ≥90%. Ciprofloxacin was purchased from Sigma-Aldrich (St Louis, MO, USA). Ciprofloxacin stock solution (5120 µg/mL) in milli Q water was prepared and stored at −30 °C.

### 4.2. Bacterial Strains and Evaluation of Minimum Inhibitory and Bactericidal Concentrations

*P. aeruginosa* 6294, 6206, Paer1, ATCC 19660 were screened for their ability to develop resistance against AMPs and ciprofloxacin at sub-MIC (one-fold below the MIC), but did not develop resistance (data not shown). *P. aeruginosa* ATCC 27853 has previously been used to examine its susceptibility to a variety of antimicrobial agents [47,48,49,50], shown to be heteroresistant to colistin [51], and used to examine the ability of *P. aeruginosa* to develop resistance to a variety of antimicrobial agents [52,53,54]. This strain’s ability to develop resistance against AMPs and ciprofloxacin at sub-MICs (one-fold below the MIC) was examined as previously described [55].

The minimum inhibitory concentration (MIC) and minimum bactericidal concentrations (MBC) of ciprofloxacin were determined using a standard broth microdilution method of the Clinical Laboratory and Standard Institute (CLSI) and a modified version of the CLSI broth microdilution method was used to determine the MIC of antimicrobial peptides [56] in Muller Hinton broth (MHB; Oxoid, Basingstoke, UK). The MIC was set as the lowest concentration of peptides that reduced bacterial growth by ≥90% while the MBC was set as the lowest concentration of peptides that reduced bacterial growth by >99.99% following enumeration of live bacteria by plate counts compared to bacteria grown in the absence of antimicrobials.

### 4.3. Growth Curve and Resistance Development at Sub-MIC of Antimicrobials

An aliquot (100 µL) of an overnight culture (6 × 10^5^ CFU ml^−1^) of bacteria was added to an equal volume of each antimicrobial to achieve a sub-MIC (0.5× MIC) in MHB and incubated at 37 °C with shaking at 120 rpm for 24 h. The turbidity of the bacterial suspensions was determined at OD_660nm_ over time, for 24 h. Bacteria grown in wells without antimicrobials served as positive controls for maximum bacterial growth. Serial passages of *P. aeruginosa* ATCC 27853 were performed in the presence of each antimicrobial at 0.5× MIC. After incubation for 18–24 h, cells were re-passage into fresh media containing sub-MICs of the antimicrobials. After every passage, the MIC for each antimicrobial was determined, and a new sub-MIC was used if required, due to any increase in MIC. This re-passaging was continued for 30 consecutive days.

### 4.4. Inhibition of Biofilm Formation by AMPs and Ciprofloxacin Alone or in Combination

Inhibition of biofilm formation was determined using *P. aeruginosa* ATCC 27853 that had been passaged at 0.5× MIC of ciprofloxacin for one day (sensitive cells) or 30 days (resistant cells). *P. aeruginosa* (100 µL of 6 × 10^5^ CFU/mL) was dispensed into round-bottom 96-well microtiter plates containing serial dilutions (0.5× to 4× MIC) of melimine, Mel4 or ciprofloxacin in MHB. The combined effect of melimine or Mel4 with ciprofloxacin was determined after adding equal volumes of each at their corresponding MICs. The plates were incubated at 37 °C for 24 h. Wells containing bacteria and MHB without antimicrobials acted as controls. Following incubation, the media was removed, and the wells were then carefully washed three times with HEPES buffer to remove non-adherent cells. Subsequently, biofilms were fixed with 200 µL of 99% *v*/*v* methanol for 15 min and then plates were air-dried. Finally, biofilms were stained with 200 µL of 1% *w*/*v* crystal violet dissolved in water for 5 min. Unbound crystal violet was rinsed off with 200 µL tap water and plates were inverted to air dry. Following this, the crystal violet absorbed in biofilms was solubilized in 200 µL glacial acetic acid (33% *v*/*v*), the released dye was moved to a new well, and the amount of dye released was determined spectroscopically at OD_600nm._ The degree of biofilm inhibition was determined as a percentage of the biofilm produced by the controls of bacteria with no antimicrobials using the following formula [36].

$$\begin{array}{l} \%\;biofilm\;inhibition\;of\;single\;or\;combined\;antimicrobial\\ =\frac{\left(OD_{600nm}\;of\;control\right)-\left(OD_{600nm}\;of\;individual\;or\;\left(combined\right)antimicrobials\right)}{OD_{600nm}\;of\;control}\times 100 \end{array}$$

### 4.5. Disruption of Pre-Formed Biofilms by AMPs and Ciprofloxacin Alone or in Combination

Biofilms of each bacterium were formed by adding 100 µL of *P. aeruginosa* ATCC 27853 (6 × 10^5^ CFU/mL) into round-bottom 96-well microtiter plates containing 100 µL of MHB. After incubation at 37 °C for 24 h, biofilms were treated with serially diluted peptides or ciprofloxacin or their combination in HEPES, and the plates were incubated for a further 24 h at 37 °C. Wells containing bacteria and MHB served as negative controls, while wells containing only MHB were treated as blank. The amount of biofilm was determined using crystal violet as outlined in the above.

In addition, confocal laser scanning microscopy was performed on pre-formed biofilms of 30-day ciprofloxacin passaged *P. aeruginosa* ATCC 27853 [57]. Biofilms were formed in polystyrene plates as described above but, in this case, sterile round glass coverslips had been added to the wells prior to incubation with bacteria. After formation, biofilms were treated with 200 µL of 4× MIC of melimine, Mel4 or ciprofloxacin alone or in combination at 37 °C for 24 h. After treatment, glass coverslips were removed, and biofilms were stained with Live/Dead BacLight bacterial viability kit (Invitrogen, Eugene, OR, USA), and examined by confocal microscopy (FV 1200, Olympus, Japan). The resulting data were processed using the ImageJ software version 8 (Bethesda, MD, USA).

### 4.6. Mechanistic Studies

As there was a similar antibiofilm activity of melimine and Mel4 against either 1-day or 30-day ciprofloxacin-passaged strains of *P. aeruginosa* ATCC 27853, 30-day ciprofloxacin passaged strains were selected to evaluate the mechanism of action of both the AMPs and ciprofloxacin towards bacterial cells in biofilms.

Depolarization of the bacterial cell membrane in biofilms was determined as described by Pulido et al. [58] by measuring changes in fluorescence, due to release of pre-loaded DiSC3(5) at regular intervals up to 6 h after addition of melimine, Mel4 or ciprofloxacin at 1×, 2× and 4× their respective MICs. The combined effect of peptides and ciprofloxacin was evaluated using 100 µL of melimine or Mel4 in combination with ciprofloxacin at 1×, 2× and 4× MICs. DMSO (20%; Merck, Billerica, MA, USA) was used as a positive control to achieve maximum membrane depolarization.

In addition, the supernatants of biofilms removed after incubation for 3 h and filtered through 0.22 µM pore membranes were used to measure the concentration of ATP and OD_260nm_ absorbing material as previously described, with HEPES buffer-treated samples as negative controls [59,60]. The results for the OD_260nm_ absorbing material were expressed relative to the initial OD_260nm_ of biofilms taken at 0 min. The presence of nucleic acids in supernatants was also confirmed using Sytox green (Invitrogen, Eugene, Oregon, USA). Aliquots (100 µL) of the supernatants were added to equal amounts of HEPES buffer containing Sytox green at a final concentration of 5 µM. An increase in fluorescence, due to the interaction of Sytox green with nucleic acid, was measured spectrophotometrically at an excitation wavelength of 480 nm and an emission wavelength of 523 nm.

### 4.7. Statistical Analysis

All experiments were performed in three independent assays. One-way analysis of variance (ANOVA) with Bonferroni’s correction for multiple comparisons was used to compare differences between control and antimicrobial-treated cells. The data of cell membrane depolarization were analyzed using Two-way ANOVA with Tukey’s test. A probability value of <0.05 was considered statistically significant.

## 5. Conclusions

In conclusion, *P. aeruginosa* in suspension was unable to develop resistance to melimine or Mel4 following repeated exposure to sub-inhibitory concentrations. Whilst both AMPs inhibited biofilm formation, and once *P. aeruginosa* had produced a biofilm, the cells required higher concentration (4× MIC) of melimine or Mel4 for eradication. The resistance of biofilms could be overcome by using a combination of the AMPs and ciprofloxacin, possibly as the result of the AMPs damaging the cell membrane of biofilm cells, which may have resulted in increased or facilitated uptake of ciprofloxacin. In future research fluorescently labelled ciprofloxacin will be used to examine whether the combined use of AMPs and ciprofloxacin can result in greater uptake of ciprofloxacin.

## Figures and Tables

**Table 1:** MIC and MBC of melimine, Mel4 and ciprofloxacin against *P. aeruginosa.*

Bacterial Strains	Melimine		Mel4		Ciprofloxacin	
	MIC µgmL^−1^ (µM)	MBC µgmL^−1^ (µM)	MIC µgmL^−1^ (µM)	MBC µgmL^−1^ (µM)	MIC µgmL^−1^ (µM)	MBC µgmL^−1^ (µM)
***P. aeruginosa* 6206**	250 (66.02)	500 (132.04)	62.5 (26.6)	125 (53.24)	0.25 (0.75)	0.5 (1.5)
***P. aeruginosa* 6294**	250 (66.02)	2000 (528.16)	62.5 (26.6)	250 (106.4)	0.5 (1.5)	0.5 (1.5)
***P. aeruginosa* Paer1**	250 (66.02)	500 (132.04)	250 (106.4)	500 (212.8)	0.5 (1.5)	1 (3)
***P. aeruginosa* ATCC 19660**	500 (132.04)	1000 (264.08)	62.5 (26.6)	62.5 (26.6)	1 (3)	2 (6)
***P. aeruginosa* ATCC 27853**	125 (33.01)	250 (66.02)	125 (53.24)	250 (106.48)	0.5 (1.5)	1 (3)
